# Augmenting camera images with gamma detector data: A novel approach to support sentinel lymph node biopsy

**DOI:** 10.1186/s40658-019-0245-z

**Published:** 2019-06-18

**Authors:** Peter A. von Niederhäusern, Simon Pezold, Uri Nahum, Carlo Seppi, Guillaume Nicolas, Michael Rissi, Stephan K. Haerle, Philippe C. Cattin

**Affiliations:** 1 Department of Biomedical Engineering, University of Basel, Allschwil, CH-4123 Switzerland; 2 University Hospital Basel, Radiology & Nuclear Medicine Clinic, Basel, CH-4031 Switzerland; 3 DECTRIS Ltd., Baden-Dättwil, CH-5405 Switzerland; 4 Center for Head and Neck Surgical Oncology and Reconstructive Surgery, Hirslanden Clinic, Lucerne, CH-6006 Switzerland

**Keywords:** Sentinel lymph node biopsy, Radioguided surgery, Augmented reality, Projective geometry, Multi-modality calibration

## Abstract

**Background:**

Squamous cell carcinoma in the head and neck region is one of the most widespread cancers with high morbidity. Classic treatment comprises the complete removal of the lymphatics together with the cancerous tissue. Recent studies have shown that such interventions are only required in 30% of the patients. Sentinel lymph node biopsy is an alternative method to stage the malignancy in a less invasive manner and to avoid overtreatment. In this paper, we present a novel approach that enables a future augmented reality device which improves the biopsy procedure by visual means.

**Methods:**

We propose a co-calibration scheme for axis-aligned miniature cameras with pinholes of a gamma ray collimating and sensing device and show results gained by experiments, based on a calibration target visible for both modalities.

**Results:**

Visual inspection and quantitative evaluation of the augmentation of optical camera images with gamma information are congruent with known gamma source landmarks.

**Conclusions:**

Combining a multi-pinhole collimator with axis-aligned miniature cameras to augment optical images using gamma detector data is promising. As such, our approach might be applicable for breast cancer and melanoma staging as well, which are also based on sentinel lymph node biopsy.

## Background

**Fig. 1:**
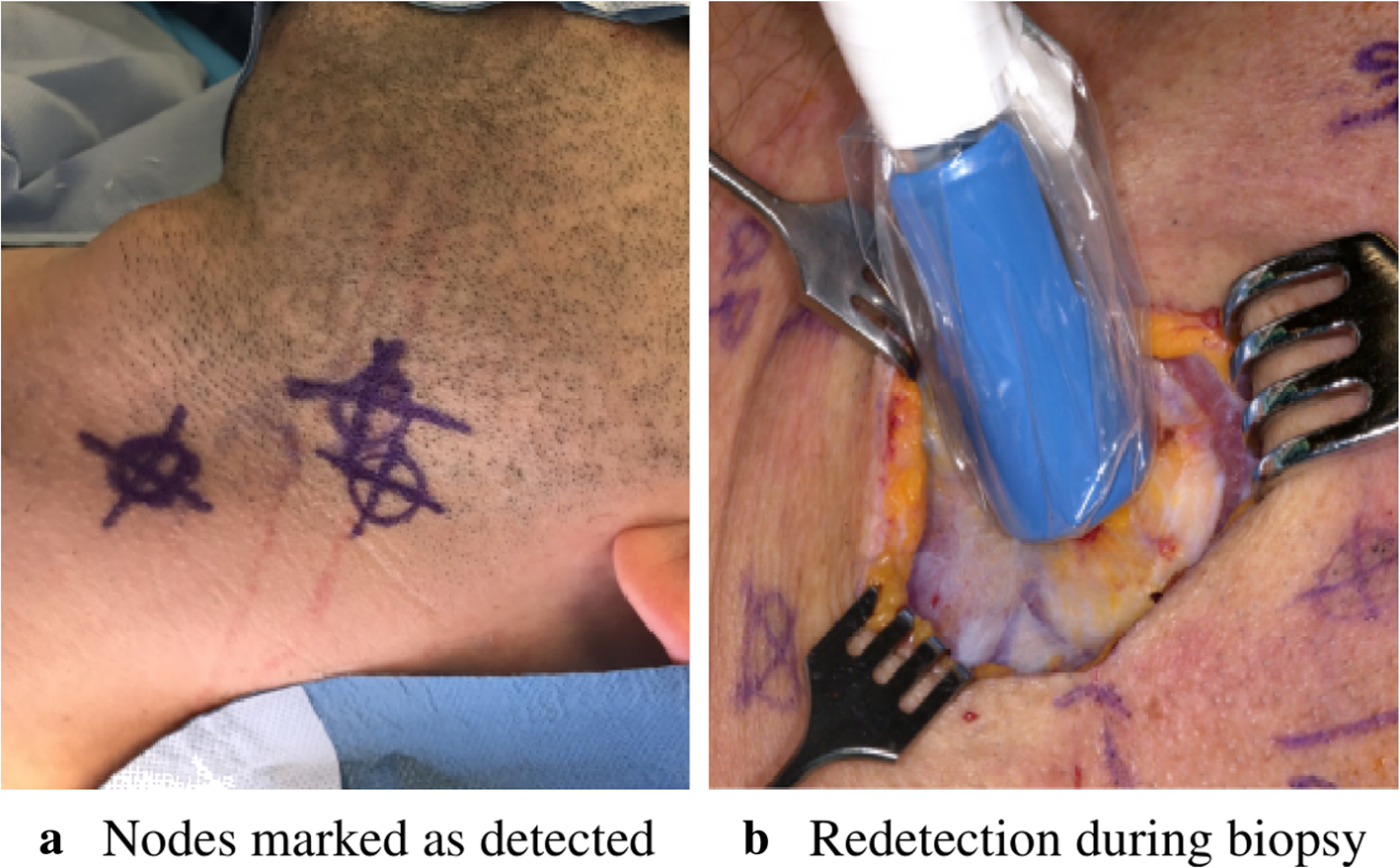
Two consecutive intervention steps of the current SNB procedure: **a** prior to the intervention, lymph nodes are localized and hand-marked accordingly. **b** As tissue is pushed away during the biopsy, the lymph nodes need to be redetected using the gamma probe

Head and neck squamous cell carcinoma (HNSCC) is one of the most prevalent cancers. Studies have shown that 70% of all HNSCC patients undergo overtreatment due to the limited accuracy of classic clinical and radiologic staging [1]. The specialist speaks of overtreatment if removal of unaffected lymph tissue is conducted. Sentinel lymph node biopsy (SNB) is the standard minimally invasive staging procedure for melanoma and breast cancer surgery and is currently validated in the domain of HNSCC treatment [2, 3]. SNB is made possible by detecting gamma radiation from treated tissue. By injecting a technetium (^99m^Tc)-based radioactive tracer, its uptake into the neighboring lymphatics can be measured by means of a gamma detector. Draining lymph nodes downstream of the tumor are amenable to acquire cancerous cells, and a biopsy of these identified so-called sentinels reveals whether spreading of the cancer has already begun [4]. If the histopathologic analysis is negative (“clinically negative neck,” i.e., cN0-neck), no neck dissection (ND) is conducted and a potential overtreatment thus avoided [1]. As SNB enables the most accurate histologic examination, it could transform HNSCC surgery to be less invasive by improving the overall staging process [5]. In the current clinical practice, a handheld gamma detection probe guides the surgeon during the biopsy by providing a one-dimensional audio-based activity indication of the tracer in the tissue or the lymphatics (Fig. 1). In order for such an intervention to succeed, the surgeon obtains additional anatomic guidance from the preoperative assessment gained by SPECT/CT imaging. However, the crude intraoperative activity measurement makes it difficult for the specialist to target sentinel lymph nodes (SLNs). They are rather small and easy to miss (≈ 5 mm in diameter). It is consensus that lymph nodes with a high radioactivity foreground-to-background signal ratio are candidate sentinels [6]. A further complication is the distinction between the tracer accumulation at the injection site and those sentinel hot spots [7]. In HNSCC staging, this problem can be circumvented as the injection site is usually away from the biopsy site as the tracer is injected into the tongue. Inaccurate SNB staging due to missing SLNs would jeopardize the health of the patient and cause a disadvantage over classic ND treatment. Given these challenges of SNB, it is evident that intraoperative image-based 2D-to-2D visualization methods improve the procedure and remove the cognitive load for the surgeon(1D-to-3D, i.e., the need to correlate audio-based activity indication with anatomic structures). 

One commercially available system for SNB, based on freehand SPECT (fhSPECT) and augmented reality (AR), is sold by the company SurgicEye (Munich, Germany) and was initially developed at TU Munich [8]. Freehand SPECT depends on a preparation and registration step to align the tracked gamma probe with the patient. Gamma activity needs to be manually re-measured every time an update for the synthetic three-dimensional model of the activity distribution is requested. Finally, this distribution model is displayed on an external monitor in the proximity of the surgeon which is not necessarily in their field of view [9]. The sequential nature of the procedure makes this approach rather involved for the operator.

Additionally, the IAEA provides an overview of current technologies and procedures of the field in the guided intraoperative scintigraphic tumor targeting (GOSTT)^1^ report.

Our proposed method, as described in the following sections, allows the operator to *directly* visualize and identify known anatomic features and to correlate them with the overlaid measured gamma activity, without manual scanning of the surgical scene. This could, in a next step, lead to a device that supports the oncologist to visually localize and compare lymph nodes, as seen on a display, with congruent high activity to indicate sentinels to excise and examine for occult tumor cells. A proposed setup of the device is to firmly attach it onto a maneuverable small platform near the patient to allow for better placement and to avoid interference with the workspace of the surgeon. In our experiments, a pack of four endoscopic CMOS cameras is placed on top of pinholes of a small form factor multi-pinhole collimator (Fig. 8b). In general, a multi-pinhole collimator has the additional benefit of increased photon gathering capabilities (sensitivity) compared to a mono-pinhole collimator. This multi-pinhole collimator is directly attached to a highly sensitive gamma detector and measurement unit. The optical axes of the cameras and the pinholes are aligned such that incident photon rays of the gamma and the optical regime are seen under the same or similar viewing angle (Fig. 8a). Thanks to the comparable opto-geometric arrangement of the cameras and the pinholes, projective geometry supports the image augmentation process, i.e., the embedding of additional information normally not seen, which is simpler and more direct than collecting and modeling a synthetic three-dimensional gamma activity distribution. Each of the four cameras can be used such that a specific augmentation with selective parameters is presented to the user.

We recently published a feasibility study that showed how an augmentation of different modalities can be done, given a miniature camera that is axis-aligned with its harboring pinhole [10]. In this current paper we build upon the former and present the accompanying theoretical principles, why axis alignment supports the augmentation process even in the presence of a depth estimate and small alignment errors (“Methods and materials” section), and more varied experimental data showing the correspondence with respect to these governing principles (“Results” section). Finally, we discuss the achieved and provide thoughts about potential improvements and future applications of the method (“Discussion” section).

## Methods and materials

This section is structured as follows: the mathematical formalism and necessary preconditions of the approach are presented in the “Foundation” section. As the hardware assembly process inevitably introduces rotations, tilts, and small offsets in the alignment of the optical cameras with respect to their harboring pinholes, a calibration scheme, given in the “Calibration” section, is needed to assess these axis alignment differences. A depth prior of the gamma activity has to be determined to further improve the augmentation: a working distance of the detector to the activity inside the patient’s lymphatics needs to be estimated. For this to be valid, we assume isotropic radiation emission. The pose parameters (the position and orientation of a camera with respect to the origin) from the calibration are reused to obviate the need for a new ad hoc pose estimation during each intervention. It is generally hard to accomplish this with sufficient accuracy in a bright lit surgical setting, especially for cameras with low-resolution sensors. Thus, supportive error minimization schemes are needed and discussed in the “Error minimization schemes” section. The technical specifications of the collimator, the miniature endoscopic CMOS cameras and the detector are given in the “Hardware” section. The augmentation algorithm of the optical camera image with gamma information, based on these principles, is explained in the Appendix.

### Foundation

**Fig. 2:**
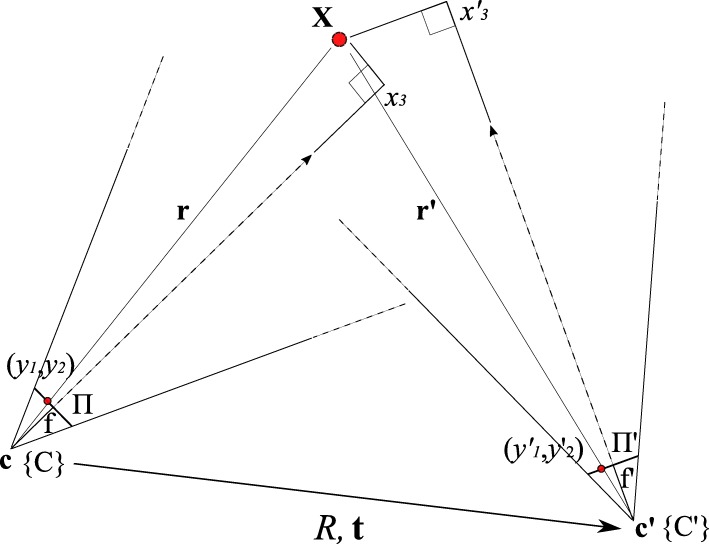
Schematic of a camera pair relation (exaggerated for illustrative purposes) with the corresponding projection of the world point **X** onto the respective image planes Π,*Π*^′^. The relative rotation *R* and translation **t** are known from calibration. The smaller *R* and **t**, the better the co-aligment between both cameras, making the overlay of their respective images possible
- Let **X** be a point in 3D space, given by **x**=(*x*_1_,*x*_2_,*x*_3_)^*T*^ in {*C*} and
  by $\mathbf {x^{\prime }}=(x_{1}^{\prime },x_{2}^{\prime },x_{3}^{\prime })^{T}$ in {*C*^′^}, respectively, where $x_{3},x_{3}^{\prime }$ give the values along the optical axes of the respective cameras (i.e., the distance or “depth”).
- Let *T* be the transformation from {*C*} to {*C*^′^}, that is
  1 $$ \left(\begin{array}{c} \mathbf{x}^{\prime}\\ 1 \end{array}\right)=T\,\left(\begin{array}{c} \mathbf{x}\\ 1 \end{array}\right),  $$
  where
  2 $$ T=\left[\begin{array}{cc} R & \mathbf{t}\\ 0 & 1 \end{array}\right]  $$
  with the 3×3 rotational part *R*=[*ρ*_1_|*ρ*_2_|*ρ*_3_]^*T*^ (i.e., *ρ*_._ form the rows (!) of *R*) and translational part **t**=(*t*_1_,*t*_2_,*t*_3_)^*T*^, both known from the calibration step (“Calibration” section).
- Let **y**=(*y*_1_,*y*_2_)^*T*^ and $\mathbf {y}^{\prime }=(y_{1}^{\prime },y_{2}^{\prime })^{T}$ be the points on the respective image planes in the units of the world coordinates (i.e., mm, units of the calibration target) and let
  3 $$ \tilde{\mathbf{y}}=\frac{1}{f}\thinspace\mathbf{y}\quad\text{and}\quad\tilde{\mathbf{y}}^{\prime}=\frac{1}{f^{\prime}}\thinspace\mathbf{y}^{\prime}  $$
  with $\tilde {\mathbf {y}}=(\tilde {y}_{1},\tilde {y}_{2})^{T}$ and ${\tilde {\mathbf {y}}^{\prime }}=\left (\tilde {y}_{1}^{\prime },\tilde {y}_{2}^{\prime }\right)^{T}$ be these very plane coordinates, normalized by the respective focal lengths *f*,*f*^′^, both intrinsic parameters known.
- Let *I*_*d*_ be the *d*×*d* identity matrix.

The goal is to overlay a point that is seen by one pinhole camera, in our case the optical camera, with information from the other pinhole camera, in our case the gamma camera, when the configurations of the two cameras are known but not their distances to the projected world point, whose coordinates are given in relation to the origin. First, some definitions (*cf.* Fig. 2):

- Let {*C*} and {*C*^′^} be the coordinate systems or frames of two cameras.

Making use of the *intercept theorem*, we observe that for camera {*C*}, the following equalities hold (assuming *f*>0,*x*_3_>0):

4 $$\begin{array}{*{20}l} \left(\begin{array}{c} y_{1}\\ y_{2}\\ f \end{array}\right) & =\frac{f}{x_{3}}\, \begin{array}{c} \mathbf{x} \end{array} \end{array} $$

5 $$\begin{array}{*{20}l} \Longleftrightarrow\left(\begin{array}{c} \tilde{y}_{1}\\ \tilde{y}_{2}\\ 1 \end{array}\right) & =\frac{1}{x_{3}}\, \begin{array}{c} \mathbf{x} \end{array}, \end{array} $$

6 $$\begin{array}{*{20}l} \Longleftrightarrow x_{3}\,\left(\begin{array}{c} \tilde{y}_{1}\\ \tilde{y}_{2}\\ 1 \end{array}\right) & = \begin{array}{c} \mathbf{x}\\ \end{array}, \end{array} $$

and likewise for {*C*^′^} (assuming $f^{\prime }>0,x_{3}^{\prime }>0$):

7 $$ \left(\begin{array}{c} \tilde{y}_{1}^{\prime}\\ \tilde{y}_{2}^{\prime}\\ 1 \end{array}\right)=\frac{1}{x_{3}^{\prime}}\, \begin{array}{c} \mathbf{x}^{\prime} \end{array}.  $$

Let us now assume that we measure $\tilde {\mathbf {y}}$(or rather: **y**, which immediately gives us $\tilde {\mathbf {y}}$) for the point **X** in {*C*}’s camera plane and that we want to determine the respective$\tilde {\mathbf {y}}^{\prime }$ in {*C*^′^}’s camera plane, solely based on that measurement$\tilde {\mathbf {y}}$. Let us therefore express {*C*^′^}’s world coordinates in terms of {*C*}—which we can do by making use of Eq. (1)—and insert in Eq. (7), which gives us

8 $$ \left(\begin{array}{c} \tilde{y}_{1}^{\prime}\\ \tilde{y}_{2}^{\prime}\\ 1\\ 1 \end{array}\right)=\left[\begin{array}{cc} \frac{1}{x_{3}^{\prime}}\,I_{3} & 0\\ 0 & 1 \end{array}\right]\,T\,\left(\begin{array}{c} \mathbf{x}\\ 1 \end{array}\right).  $$

Now let us insert Eq. (6), that is, {*C*}’s image plane coordinates, on the right:

9 $$\begin{array}{*{20}l}  \left(\begin{array}{c} \tilde{y}_{1}^{\prime}\\ \tilde{y}_{2}^{\prime}\\ 1\\ 1 \end{array}\right) & = S_{c'}\, T\, S_{c}\, \left(\begin{array}{c} \tilde{y}_{1}\\ \tilde{y}_{2}\\ 1\\ 1 \end{array}\right) \end{array} $$

with

10 $$\begin{array}{*{20}l} S_{c^{\prime}} = \left[\begin{array}{cc} \frac{1}{x_{3}^{\prime}}\,I_{3} & 0\\ 0 & 1 \end{array}\right], \ S_{c} = \left[\begin{array}{cc} x_{3}\,I_{3} & 0\\ 0 & 1 \end{array}\right]. \end{array} $$

We denote $S_{c^{\prime }}$ and *S*_*c*_*scaling matrices* (*cf.* Eq. (7)) and above Eq. (9) can equivalently be written as

11 $$ \tilde{\mathbf{y}}^{\prime}=\frac{x_{3}}{x_{3}^{\prime}}\,R\,\tilde{\mathbf{y}}+\frac{\mathbf{t}}{x_{3}^{\prime}}.  $$

Here, we realize that we can express *x*_3_ in terms of $x_{3}^{\prime }$ via Eqs. (1)+(6). In particular, we may write

12 $$\begin{array}{*{20}l}  x_{3}^{\prime} & =x_{3}\,\rho_{3}\cdot\tilde{\mathbf{y}}+t_{3} \end{array} $$

13 $$\begin{array}{*{20}l} \Longleftrightarrow x_{3} & =\frac{x_{3}^{\prime}-t_{3}}{\rho_{3}\cdot\tilde{\mathbf{y}}},  \end{array} $$

which enables us to rewrite Eq. (11) as

14 $$\begin{array}{*{20}l} \tilde{\mathbf{y}}^{\prime} & =\frac{1-\nicefrac{t_{3}}{x_{3}^{\prime}}}{\rho_{3}\cdot\tilde{\mathbf{y}}}\,R\,\tilde{\mathbf{y}}+\frac{\mathbf{t}}{x_{3}^{\prime}} \end{array} $$

15 $$\begin{array}{*{20}l} \Longleftrightarrow\tilde{\mathbf{y}}^{\prime} & =\frac{1}{\rho_{3}\cdot\tilde{\mathbf{y}}}\,R\,\tilde{\mathbf{y}}+\frac{1}{x_{3}^{\prime}}\,\left(\frac{-t_{3}}{\rho_{3}\cdot\tilde{\mathbf{y}}} \,R\,\tilde{\mathbf{y}}+\mathbf{t}\right). \end{array} $$

#### Approximating $\tilde {\mathbf {y}}^{\prime }$ without knowing $x_{3}^{\prime }$

In Eq. (15), only $x_{3}^{\prime }$ remains unknown, which, in turn, only affects the right term of the equation. Assuming the right term was zero, we could exactly express $\tilde {\mathbf {y}}^{\prime }$ in terms of $\tilde {\mathbf {y}}$, namely

16 $$ \tilde{\mathbf{y}}^{\prime}=\frac{1}{\rho_{3}\cdot\tilde{\mathbf{y}}}\,R\,\tilde{\mathbf{y}}.  $$

For approximating $\tilde {\mathbf {y}}^{\prime }$ without knowing $x_{3}^{\prime }$, we can thus try to *change our camera setup* so that we minimize the right term of Eq. (15), making Eq. (16) a good approximation of $\tilde {\mathbf {y}}^{\prime }$. We have two options for this:

1. Simultaneously *moving the cameras away* from the imaged point *X* in the direction of {*C*^′^}’s optical axis or vice versa, leaving *T* unchanged, thus
  17 $$ x_{3}^{\prime}\rightarrow\infty\Longleftrightarrow\nicefrac{1}{x_{3}^{\prime}}\rightarrow0\Longrightarrow\frac{1}{x_{3}^{\prime}}\, \left(\frac{-t_{3}}{\rho_{3}\cdot\tilde{\mathbf{y}}}\,R\,\tilde{\mathbf{y}}+\mathbf{t}\right)\rightarrow\mathbf{0}.  $$
2. *Minimizing the distance |***t***| between the cameras* while keeping the distance $x_{3}^{\prime }$, by moving {*C*} as close as possible to {*C*^′^}, thus
  $$\left|\mathbf{t}\right|\rightarrow0\Longrightarrow\left[-t_{3}\rightarrow0\text{ and }\mathbf{t}\rightarrow\mathbf{0}\right]\Longrightarrow\frac{1}{x_{3}^{\prime}}\,\left(\frac{-t_{3}}{\rho_{3}\cdot \tilde{\mathbf{y}}}\,R\,\tilde{\mathbf{y}}+\mathbf{t}\right)\rightarrow\mathbf{0}. $$

We have a third option to get a good approximation for $\tilde {\mathbf {y}}^{\prime }$ without knowing $x_{3}^{\prime }$, even without changing our camera setup: in Eq. (15), we can *use a reasonable estimate*$\hat {x}_{3}^{\prime }$ in place of $x_{3}^{\prime }$ (i.e. $\hat {x}_{3}^{\prime }\approx x_{3}^{\prime }$), giving us an estimate $\hat {\mathbf {y}}^{\prime }$ for $\tilde {\mathbf {y}}^{\prime }$, namely

18 $$ \hat{\mathbf{y}}^{\prime}=\frac{1}{\rho_{3}\cdot\tilde{\mathbf{y}}}\,R\,\tilde{\mathbf{y}}+\frac{1}{\hat{x}_{3}^{\prime}}\, \left(\frac{-t_{3}}{\rho_{3}\cdot\tilde{\mathbf{y}}}\,R\,\tilde{\mathbf{y}}+\mathbf{t}\right)\approx\tilde{\mathbf{y}}^{\prime}.   $$

Note that all three options may be combined.

#### Estimation error

The estimation error $\mathbf {\epsilon }=\hat {\mathbf {y}}^{\prime }-\tilde {\mathbf {y}}^{\prime }$ is given by

19 $$ \mathbf{\epsilon}=\left(\frac{1}{\hat{x}_{3}^{\prime}}-\frac{1}{x_{3}^{\prime}}\right)\,\left(\frac{-t_{3}}{\rho_{3}\cdot \tilde{\mathbf{y}}}\,R\,\tilde{\mathbf{y}}+\mathbf{t}\right)  $$

or written for its components *ε*_*i*_ (*i*=1,2):

20 $$ \epsilon_{i}=\left(\frac{1}{\hat{x}_{3}^{\prime}}-\frac{1}{x_{3}^{\prime}}\right)\,\left(\frac{-t_{3}\,\rho_{i}\cdot \tilde{\mathbf{y}}}{\rho_{3}\cdot\tilde{\mathbf{y}}}+t_{i}\right).  $$

Note that using options 1 and 2 only or simply ignoring the right term of Eq. (15) corresponds to estimating $\hat {x}_{3}^{\prime }$ at infinity; thus, $\nicefrac {1}{\hat {x}_{3}^{\prime }}=0$ in Eqs. (19)+(20).

From Eqs. (19)+(20), we can see that the *alignment of the cameras’ optical axes* is critical for a small error: in the extreme case, when the optical axes are perpendicular, $\rho _{3}\cdot \tilde {\mathbf {y}}=0$, the error becomes unbounded.

### Calibration

**Fig. 3:**
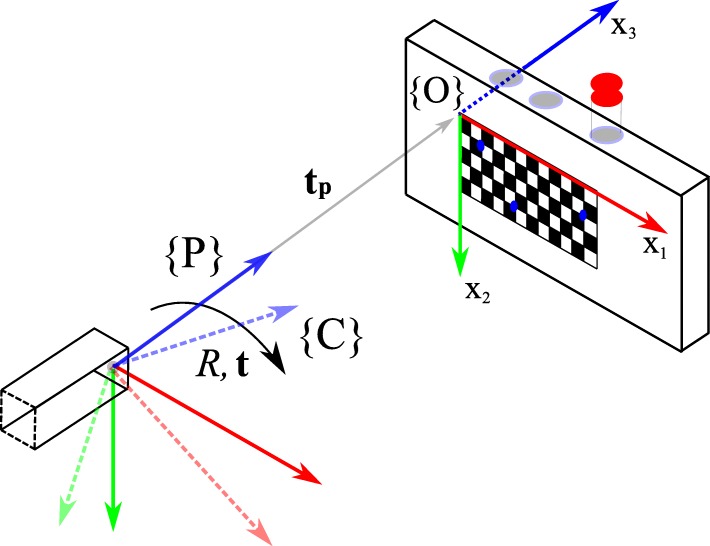
The pose of the pinhole wrt the target (solid arrows) is known. The pose of the target wrt the camera (dashed arrows) is determined by a pose estimation algorithm. Using the origin {*O*} as a pivot, we can calculate the rotation *R* and translation **t** from {*P*} to {*C*}. The tracer is contained inside the vial with the red cap. Exit pupils (blue circles) of the target are used to direct gamma radiation from the tracer for the image augmentation to assess the calibration

**Fig. 4:**
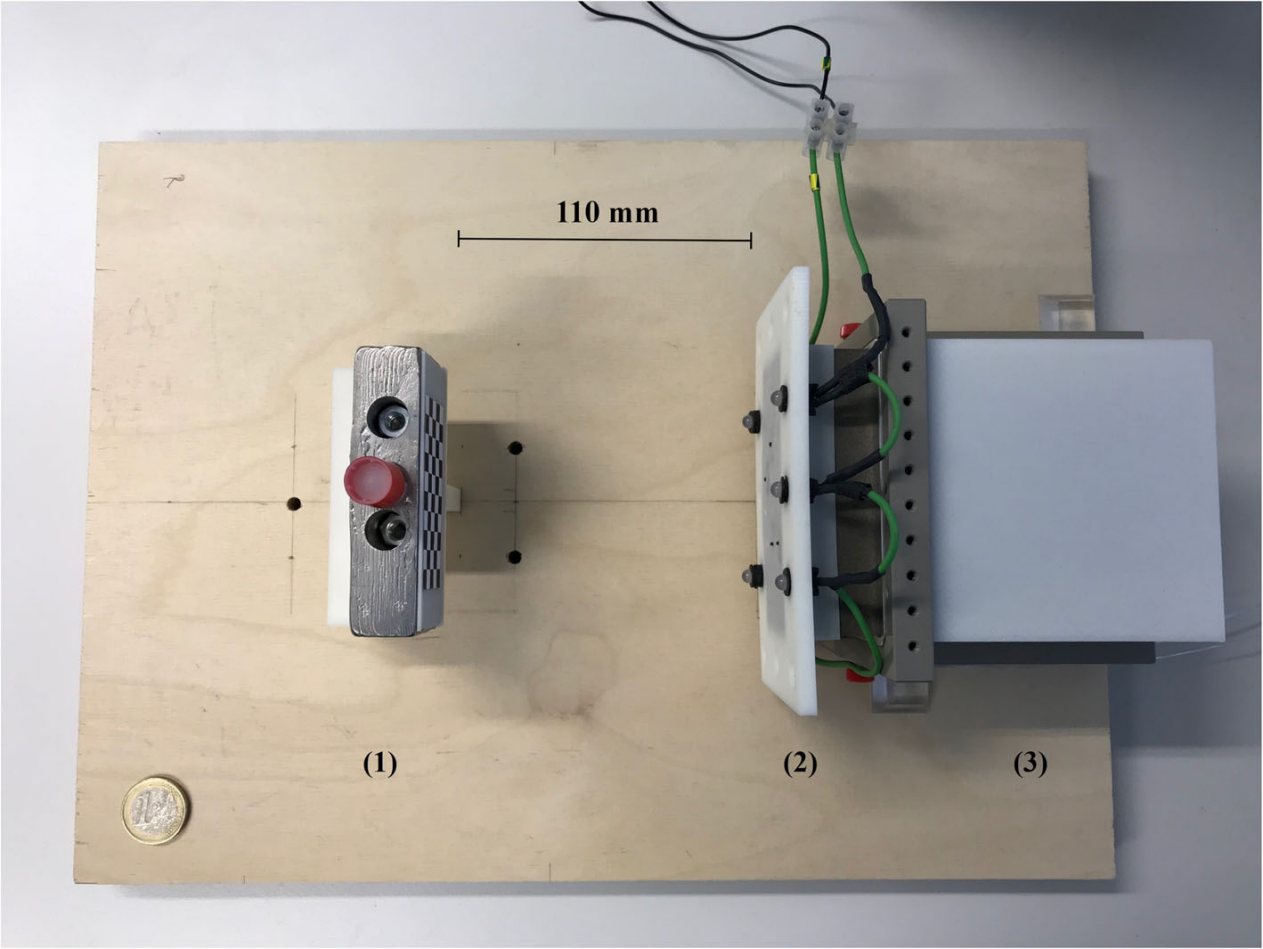
Calibration setup, viewed from the top. The stand with the calibration target and the vial (red cap) containing the tracer are placed on the left (1). The collimator with the attached camera fixation frame for the microscopic cameras and the LEDs (lighting) are to the right (2). The detector element is adjacent (3). The distance for this measurement is 110 mm from the collimator front plate to the target (*calibration default*). Not shown is the data processing unit of the detector. A 1-euro coin in the lower left serves as a scale reference
- Let {*P*} be the coordinate system of the pinhole.
- Let {*C*} be the coordinate system of the camera.
- Let *X*_*o*_ be a list of known world points of the target, given by **x**=(*x*_1_,*x*_2_,*x*_3_)^*T*^
  and ∀**x**∈*X*_*o*_: *x*_3_=0, in world coordinates.
- Let *Y*_*c*_ be the projections of *X*_*o*_, as detected by the optical camera, in camera image (buffer) coordinates.
- Let *K*_*c*_ be the given internal parameters of the optical camera (e.g., focal length).
- Let *R*_*o*_,*t*_*o*_=pose(*X*_*o*_,*Y*_*c*_,*K*_*c*_) be a pose estimation algorithm of {*O*} in {*C*}.
  This algorithm is based on the function solvePnP of the computer vision framework OpenCV [11].

We now define one pinhole camera as a purely optical camera, in the following called *camera*, and the other as a fixed pinhole of our multi-pinhole collimator, referred to simply as a *pinhole*, which form a pair, as they are physically brought together. In order to virtually realign the optical axes of such a pinhole/camera ensemble, their relative rotation *R* and translation **t** need to be determined to get *T* (Eq. (2)). We introduce definitions based on the formalism of the “Foundation” section and the calibration setup shown in Fig. 3. The actual implementation of the calibration setup can be seen in Fig. 4:

- Let {*O*} be the world coordinate system’s origin defined by a planar target. This target is the frontal plane of a Cerrobend block with exit pupils for gamma radiation to escape in a directed manner (point source).

Let us now assume that we spatially measure {*P*} solely in terms of {*O*}. This can be done as we know the relative position of the pinhole *t*_*p*_ with respect to the target origin {*O*} during calibration. As the pinhole’s image plane is perpendicularly aligned with the target (*cf.* Fig. 4), and a pinhole is part of the rigid structure of the collimator frame and thus rotation free, we are allowed to set *R*_*p*_=*I*_3_ and form the matrix *P* as

21 $$ P=\left[\begin{array}{cc} I_{3} & \mathbf{t_{p}}\\ 0 & 1 \end{array}\right],   $$

which transforms from {*O*} to {*P*}.

This is contrary to the unknown pose (i.e., position, orientation) of the optical camera with respect to {*O*}, due to rotations, tilts, and small offsets from the manufacturing process of the device. Let us assume that we measure all *X*_*o*_ and match them with the projections *Y*_*c*_. The function pose(*X*_*o*_,*Y*_*c*_,*K*_*c*_) then yields the relative measurements *R*_*c*_,*t*_*c*_ of the target in {*C*}, i.e., the mapping from {*O*} to {*C*}. In analogy to Eq. (21), we obtain the matrix *C* as

22 $$ C=\left[\begin{array}{cc} R_{c} & \mathbf{t_{c}}\\ 0 & 1 \end{array}\right].  $$

To get the complete transformation from {*P*} to {*C*}, we simply write

23 $$ R, \mathbf{t} = T = C P^{-1}.   $$

With *T*, we have determined the necessary transformation to map a world point from the pinhole coordinate system to the camera coordinate system.

In order to validate the correct transformation and mapping of gamma activity onto the optical camera image, the calibration target is equipped with bore holes for inserting vials with tracer material. As the target block is based on a Cerrobend alloy, horizontal exit pupils allow the gamma radiation from the tracer to escape towards the detector in a point-like fashion (*cf.* Fig. 3). The generated point source activity images or patches can then be compared with the known locations of the exit pupils in the optical image to visually assess the augmentation and therefore the quality of the calibration. In the “Error quantificationErrorquantification” section, we propose a quantitative approach that is shown in the “Results” section.

### Error minimization schemes

Recall the goal to overlay optical camera images with gamma activity from the detector. These two modalities must therefore be joined. If for both the camera and the pinhole their spatial relation is known, and they identify the *same* projected world point (target), we know how to transfer information from one to the other (Fig. 2). This corresponds to a homography formulation between the two sensors. On the other hand, the placement of the sensors can be chosen freely, given again that their relative spatial relation is determined, *if and only if* we knew exactly the distance(s) from either sensor to the target. This corresponds to solving Eqs. (12), (13), and by consequence Eq. (11) and is a more restrictive formulation of the above general homography. Neither of the two methods can be fully applied in our case by nature of the different modalities of the sensors. We therefore introduce axis alignment as a restriction.

#### Axis alignment

**Fig. 5:**
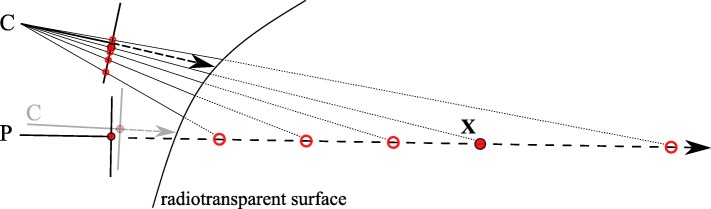
An active source (**X**) is projected along the view axis of the pinhole (P). By not knowing the true depth, any of some possible positions (circles) could be projected onto the image plane of P. This ambiguity cannot be resolved to correctly augment the image produced by the optical camera (C) with information from P. However, by moving C as close as possible to P (light gray), their respective projections converge. Adding a depth estimate improves the accuracy of the image augmentation further. Note the differences between the mapped projections of the image plane of C. We observe that with increasing distance these differences decrease, yielding a decaying error curve (*cf.* the “Error quantification” section)

In the “Foundation” section, we propose three possible options to control the augmentation error *ε*, and we conclude that axis alignment is the most viable one: moving the camera/pinhole pair away too far from the target decreases the probability of gamma photons, governed by the inverse square law, to reach the collimator and to trigger a signal on the detector. Furthermore, the focal length of the camera does only allow for a certain distance range to produce reasonably sharp images. As there is *neither* a unique target identification possible (for a potential homography), given the different modalities of the sensors, nor an *exact* distance measurement available, axis alignment helps to reduce the augmentation error. This is even more important if the activity does not coincide with the optical axes. A good depth estimate compensates for small misalignments ofthe axes (Fig. 5).

#### New scaling matrices

Our approach needs to be flexible enough to handle variations in the placement of the device, thus also the camera/pinhole pairs, in relation to the radioactive source.

In case the distance to the target is identical to the calibration setup, no new depth estimation is required. However, in a real-world scenario, this will most likely be different; here, it depends also on the depth of the lymph nodes to be detected. From the calibration step (“Calibration” section), we get a relative transform *T* from {*P*} to {*C*} which is valid as long as the camera and the pinhole are not moved or rotated with respect to each other. This can be avoided by mechanical constraints. As for the augmentation to remain valid in a new setup, we must move the pinhole and the camera such that *T* stays the same. We adjust the scaling matrices of Eq. (9) with the depth estimate *x*_3_, and $x^{\prime }_{3}$determined from Eq. (11).

We consider an operative distance range of 90–130 mm reasonable. Closer distances increase the photon pressure on the collimator while farther away targets no longer provide enough gamma radiation and incur the performance of the optical camera due to the fixed focal length. These distances also allow the surgeon to operate their biopsy tools without disturbing the confined workspace of the surgical scene further. In the “Results” section, we also show results for distances of 150 mm which are used to test the performance of the method at the system boundaries.

#### Error quantification

**Fig. 6:**
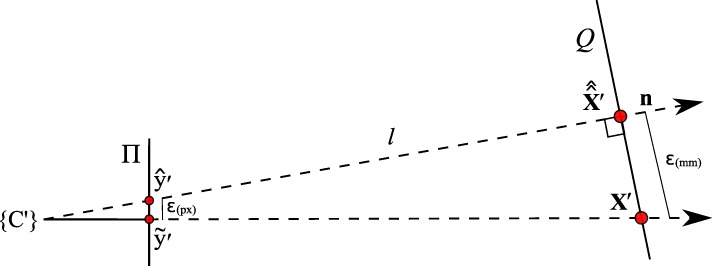
The camera on the left projects a known world point (*X*^′^) onto its image plane ($\tilde {\mathbf {y}'}$). The difference *ε*_(px)_ of the augmentation ($\hat {\mathbf {y}'}$), based on a depth estimate, is evaluated on the image plane (Π). The difference *ε*_(mm)_ of its reprojection ($\hat {\hat {\mathbf {X}}'}$) is evaluated on a target plane (*Q*), given in world units (mm)

**Fig. 7:**
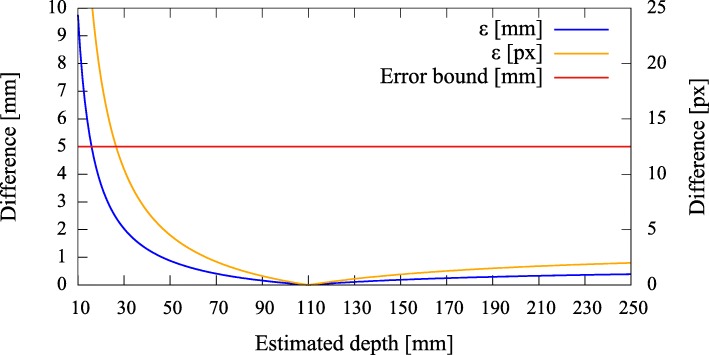
Simulated disparities *ε* in millimeter and pixels for different estimated depths, given zero camera rotation and a translation of **t**=(0.5,0.5,3.0)^*T*^ mm with respect to the pinhole. The camera is not allowed to move more thanks to constructive constraints. A minimal error is reached where our depth expectation of 110 mm matches the actual depth of the source
- Let $\tilde {\mathbf {y}^{\prime }}$ be the projection of the world point onto the image plane.
- Let $\hat {\mathbf {y}^{\prime }}$ be the augmentation, based on a depth estimate.
- Let $\hat {\hat {\mathbf {X}}}^{\prime }$ be the reprojection of $\hat {\mathbf {y}^{\prime }}$ to yield the virtual source location.
- Let {*C*^′^} be the coordinate system of the camera.

To be able to give a quantitative measure, we first modeled the effect of axis misalignment in the presence of a depth estimate (*cf.* Fig. 6). Depending on the pose and location of the camera with respect to the pinhole, and the accuracy of the estimate, the differences between the *known* projection of the world point and its *estimated* projection and reprojection are evaluated each using the *L*_2_ norm to give rise to the metrics (errors) *ε*_(px)_ and *ε*_(mm)_ (Fig. 7). The error *ε*_(px)_ is calculated as the disparity between the *known* projection of a world point (i.e., the center of the exit pupil) onto the camera sensor and the transformation of that world point (i.e., the center of the activity blob) from the pinhole sensor onto the camera sensor according to Eq. (19) in pixel space. Furthermore, as it is relevant to the surgeon to know how far off from the actual lymph node they will initially place and drive the biopsy tools, the *estimated* augmentation is reprojected and compared with the world point in world units (mm) and its error expressed as *ε*_(mm)_. Note that in Fig. 6 we omit the drawing of the pinhole and show the already transformed activities. To calculate this reprojection, we proceed as follows and use the notation from thesections above.

- Let *X*^′^ be the known world point (true source location).

The augmentation $\hat {\mathbf {y}^{\prime }}$ is calculated according to Eq. (18). To get $\hat {\hat {\mathbf {X}}}^{\prime }$, we find a plane *Q* whose normal vector **n**=(*n*_1_,*n*_2_,1)^*T*^ is parallel to the line *l* (the reprojection) through the origin of {*C*^′^} and $\hat {\mathbf {y}^{\prime }}$. We then find the intersection of this line with the plane to get the virtual source location in the coordinate system defined by {*C*^′^}. The plane *Q* is defined as

24 $$ Q: \left(\begin{array}{c} p_{1} - x_{1}^{\prime}\\ p_{2} - x_{2}^{\prime}\\ p_{3} - x_{3}^{\prime} \end{array}\right) \cdot \left(\begin{array}{cc} n_{1}\\ n_{2}\\ 1 \end{array}\right) = \left(\begin{array}{c} p_{1} - x_{1}^{\prime}\\ p_{2} - x_{2}^{\prime}\\ p_{3} - x_{3}^{\prime} \end{array}\right) \cdot \left(\begin{array}{cc} \hat{y}_{1}^{\prime}\\ \hat{y}_{2}^{\prime}\\ 1 \end{array}\right) = 0  $$

where **p**=(*p*_1_,*p*_2_,*p*_3_)^*T*^ are points on the plane.

The line *l* through the origin of {*C*^′^} and $\hat {\mathbf {y}^{\prime }}$ is given by

25 $$ l: \mathrm{C'}_{o} + \lambda \left(\begin{array}{cc} \hat{y}_{1}^{\prime}\\ \hat{y}_{2}^{\prime}\\ 1 \end{array}\right) = \lambda \left(\begin{array}{cc} \hat{y}_{1}^{\prime}\\ \hat{y}_{2}^{\prime}\\ 1 \end{array}\right).  $$

To get $\hat {\hat {\mathbf {X}}}^{\prime }$, we need to find *λ* such that

26 $$ \left(\begin{array}{cc} \lambda\hat{y}_{1}^{\prime} - x_{1}^{\prime}\\ \lambda\hat{y}_{2}^{\prime} - x_{2}^{\prime}\\ \lambda~1 - x_{3}^{\prime} \end{array}\right) \cdot \left(\begin{array}{cc} \hat{y}_{1}^{\prime}\\ \hat{y}_{2}^{\prime}\\ 1 \end{array}\right) = 0 \Leftrightarrow \lambda \left\lVert\left(\begin{array}{cc} \hat{y}_{1}^{\prime}\\ \hat{y}_{2}^{\prime}\\ 1 \end{array}\right)\right\rVert^{2} = \left(\begin{array}{cc} x_{1}^{\prime}\\ x_{2}^{\prime}\\ x_{3}^{\prime}\\ \end{array}\right) \cdot \left(\begin{array}{cc} \hat{y}_{1}^{\prime}\\ \hat{y}_{2}^{\prime}\\ 1 \end{array}\right) \Leftrightarrow \lambda = \frac{\mathbf{X^{\prime}} \cdot \hat{\mathbf{Y}^{\prime}} }{\left\lVert\hat{\mathbf{Y}^{\prime}}\right\rVert^{2}}  $$

with $\hat {\mathbf {Y}^{\prime }} = ({\hat {y}_{1}^{\prime }}, {\hat {y}_{2}^{\prime }}, 1)^{T}$.

The coordinates of $\hat {\hat {\mathbf {X}}}^{\prime }$ in the coordinate system of {*C*^′^} are thus given by

27 $$ \hat{\hat{\mathbf{X}}}^{\prime}=\lambda \hat{\mathbf{Y}^{\prime}} = \frac{\mathbf{X^{\prime}} \cdot \hat{\mathbf{Y}^{\prime}}}{\left\lVert\hat{\mathbf{Y}^{\prime}}\right\rVert^{2}}\hat{\mathbf{Y}^{\prime}}.  $$

Error curves for varying depth estimates *x* can then be constructed. We also introduce an upper error bound to assess *ε*. As the resulting augmentation needs to be as close as possible to the center of the true source location (i.e., a lymph node, diameter ≈ 5 mm), we do not tolerate augmentation errors larger than 5 mm (Fig. 7). Furthermore, as the error can only be properly evaluated during calibration, the curves need to be read as an expected augmentation error with respect to the estimated (unknown) depth.

### Hardware

**Fig. 8:**
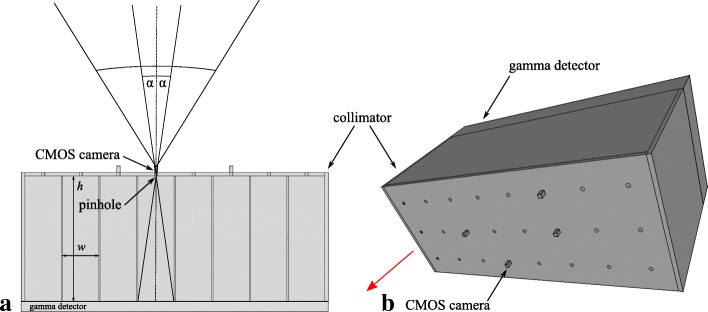
**a** Single pinhole of our multi-pinhole collimator, its field of view (2 × *α*) defined by width *w* and height *h*. The wider field of view of the aligned camera is drawn in comparison. **b** Rendering of the micro camera placement layout with respect to the pinholes (camera fixation frame not shown). The collimator is pointing towards the source (red arrow)

**Fig. 9:**
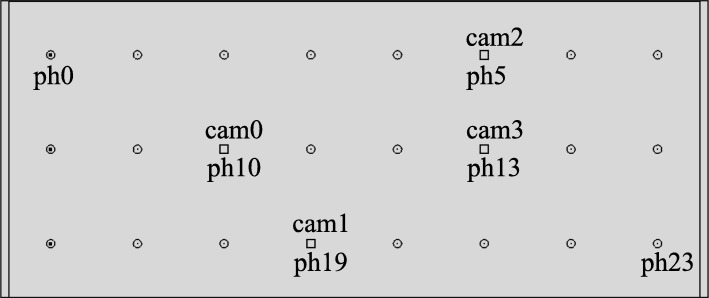
Front view of the collimator (rendering). The following camera/pinhole pair layout and naming scheme is used: pair 1 (cam0/ph10), pair 2 (cam1/ph19), pair 3 (cam2/ph5), and pair 4 (cam3/ph13). The layout of the cameras is chosen such that the coverage of the target shows some variability

The design and layout of our multi-pinhole collimator are shown in Figs. 8 and 9. This collimator is a tungsten-based device with a specific field of view, given the width and length of its pinhole compartments (Fig. 18). Each such evaluated compartment of a camera/pinhole pair is marked on the gamma sensor image in the “Results” section accordingly. The thickness of the front plate (1 mm) and the length of the septa (compartment walls, 35 mm) are calculated such that the probability of background photons to penetrate the shields is at most 5%. The dimensions of the collimator frame are 86 mm in width, 36 mm in height, and 37 mm in depth. Its weight is 300 g.

In this study, we used endoscopic cameras (model NanEye, AMS AG, Premstätten, Austria) that measure 1 mm × 1 mm × 1.7 mm in width, depth, and height, respectively. Their resolution is 250×250 pixels with a pixel size of 3 *μ*m×3 *μ*m, and thus, an aspect ratio of 1:1. The effective focal length is 660 *μ*m. The built-in optics are wide-angle lenses with an *f*-number of 2.7, an aperture of 244 *μ*m, and an optimal focus range of 8–75 mm. The cameras are mounted and fixed on an 3D printed frame (ABS) matching the pinholes of the collimator. Thanks to this mounting, the cameras are mechanically constrained such that their lateral movement cannot exceed 0.5 mm and the distance to the pinholes is kept at a maximum of 3.0 mm (*cf.* Fig. 4). As the optical cameras are exposed to high energy photons, a deteriorate effect might be expected. However, we did not observe any negative impact on the performance of the sensors over the course of our experiments.

Our industrial collaborator DECTRIS (Baden-Dättwil, Switzerland) provided us with a 2D gamma detector prototype with a native resolution of 487×195 pixels and a pixel size of 172 *μ*m×172 *μ*m. The detector technology of DECTRIS is based on Hybrid Photon Counting (HPC) and cadmium telluride (CdTe) sensor material [12]. Its quantum efficiency (QE) at 140.5 keV is 31% [13]. A high QE is crucial as only ≈ 1% or less of the injected tracer activity arrives in the lymphatics. Its dimensions are 110 mm in width, 83 mm in height, and 109 mm in depth. Its weight is 1494 g.

The overall dimensions of the combined device (collimator, gamma detector) are 110 mm in width, 83 mm in height, and 126 mm in depth.

For the experiments (*cf.* “Results” section), the cameras and pinholes are initially co-calibrated at a distance of 110 mm to yield *T* acc. to Eq. (23), using a checkerboard with tiles 6 mm × 6 mm for automatic pose estimation (“Calibration” section). Each gamma activity blob is pre-processed using a truncated Gaussian blur filter (*σ*=2, kernel size 3×3) and a minimum threshold (both operator adjustable parameters) to improve the visualization. This step is needed as the background signal from unwanted gamma photons (scatterers) is spread over the sensor area and impairs the augmentation.

## Results

The figures in this section show different augmentation results during calibration and in situ measurements. The activity blobs are colored based on a heat map according to their radiation intensity; high photon counts yield brighter colors. All measurements show results from one gamma source (vial) with an activity of ≈ 60 MBq. The vial is variably positioned at three corresponding exit pupils of the calibration target. The detector exposure time is indicated for each figure. All camera/pinhole pairs are shown for tracer positions suitable for their respective field of view; we refer to each pair according to the naming scheme of Fig. 9.

**Fig. 10:**
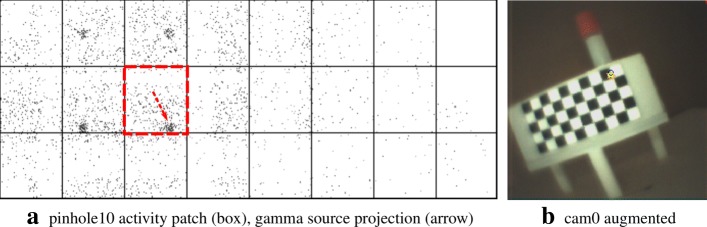
Pair 1, known source distance 90 mm (*x*_3_), acquisition time 16 s. **a** Activity image. **b** The activity accumulation (orange) of the source near the exit pupil (blue circle) of the Cerrobend ^TM^ block is shown

**Fig. 11:**
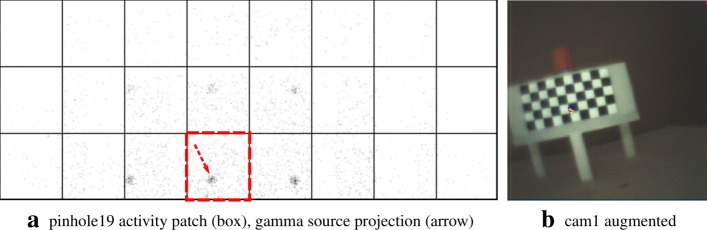
Pair 2, known source distance 110 mm (*x*_3_), acquisition time 8 s. **a** Activity image. **b** Gamma rays emanate near the exit pupil

**Fig. 12:**
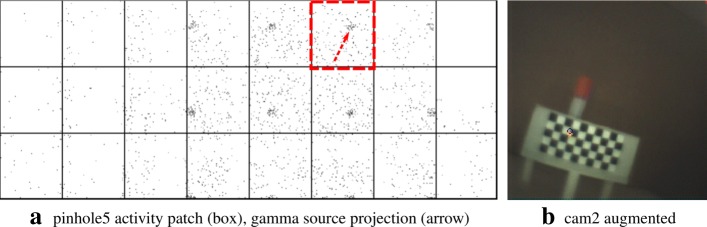
Pair 3, known source distance 130 mm (*x*_3_), acquisition time 16 s. **a** Activity image. **b** The exit pupil and the activity blob show slight discrepancies

**Fig. 13:**
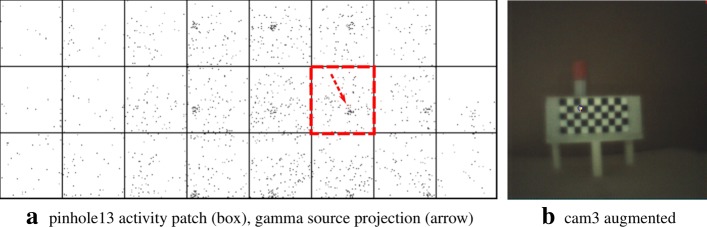
Pair 4, known source distance 150 mm (*x*_3_), acquisition time 16 s. **a** Activity image. **b** The exit pupil and the activity blob match almost exactly

**Fig. 14:**
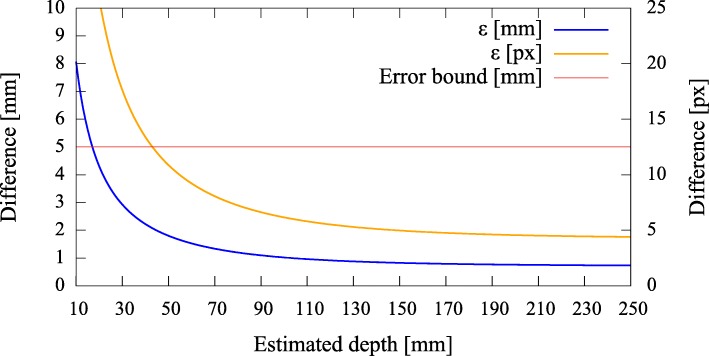
Pair 1, true source distance 90mm (*x*_3_), initial camera distance 90.8 mm (*x*^′^_3_). The curves *ε*_(mm)_,*ε*_(px)_ indicate that we are below the maximally allowed error in case the stored initial calibration is used (no automatic pose estimation). However, no apparent minimum is reached in the range of the depth estimates. Depending on the quality of the initial co-calibration at 110 mm, such effects are to be expected

**Fig. 15:**
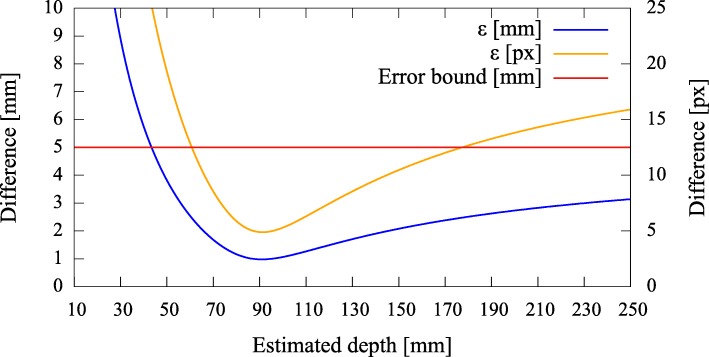
Pair 2, true source distance 110 mm (*x*_3_), initial camera distance 109.8 mm (*x*^′^_3_). Without automatic pose estimation, the error is lowest at the apparent depth of ≈ 90 mm

**Fig. 16:**
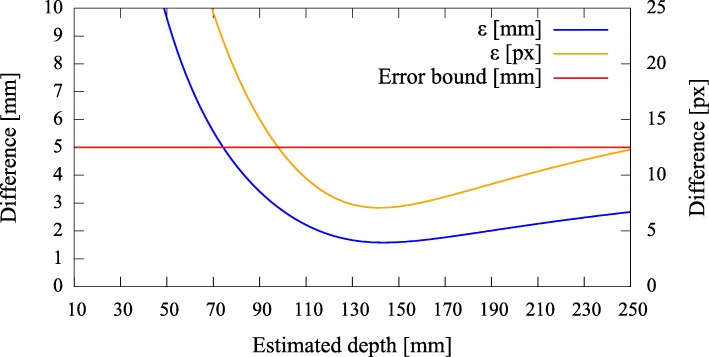
Pair 3, true source distance 130 mm (*x*_3_), initial camera distance 124.9 mm (*x*^′^_3_). The error curves show their minima at ≈ 140 mm

**Fig. 17:**
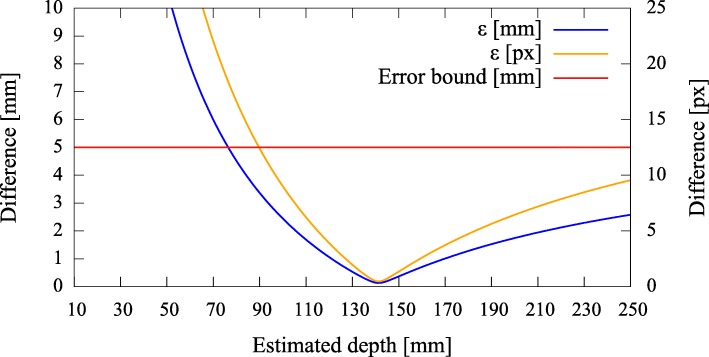
Pair 4, true source distance 150 mm (*x*_3_), initial camera distance 150.3 mm (*x*^′^_3_). The augmentation error is well below the acceptable error, even if the minimum error is reached at ≈ 140 mm

In Figs. 10, 11, 12, and 13, we present different detector activity images with associated pinhole (ph) patches (left) and corresponding augmentation results (right) based on automatic pose estimation. The respective exit pupil of the target is represented as a blue circle (unscaled for better visibility) with radius 1.5 mm, and the known distance (*x*_3_) from the pinhole to the target/source is indicated accordingly. These visualizations help to assess the calibration quality. In Figs. 14, 15, 16, and 17, the generated error curves *ε*_(mm)_,*ε*_(px)_ (disparities) are based on the stored calibration from the previous figures, without automatic pose estimation and using depth estimates in the range 10–250 mm. Based on the true initial source distance (*x*_3_), the initial camera distance ($x^{\prime }_{3}$, *cf.* Eq. (12)) is also given. These curves allow a quantitative evaluation of the estimate as well as the initial co-calibration. In our case, the relevant curve is given by *ε*_(mm)_ (*cf.* “Error quantification” section).

In Fig. 19, we reuse the stored calibration parameters on a target with a different exit pupil layout compared to the calibration target and without a pattern for pose estimation. However, the distances are known (indicated). The exit pupils with a smaller radius of 0.5 mm are visible as darkish spots. Activities are shown at or near the exit pupils. No further visual indication is given, and the error plots omitted. Figure 20a, b, and c represent images with augmentation errors. The first two images undergo augmentation with a deliberately wrong depth estimate to show the effect. The consequence of insufficient thresholding or filtering to truly identify a landmark feature is shown as a pathologic case in the third image.

**Fig. 18:**
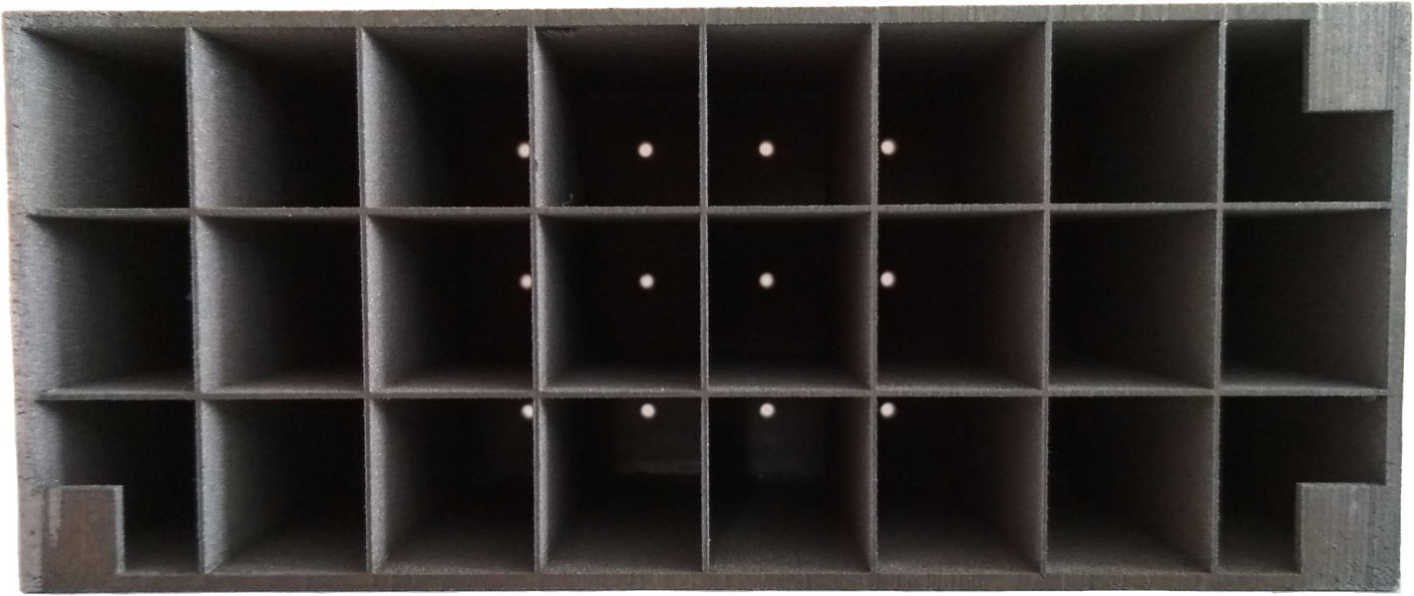
Backside view of the real collimator. Each pinhole yields an activity patch according to its compartment. These compartments define the field of view of the pinholes. Some light-passing pinholes can be seen in the middle

**Fig. 19:**
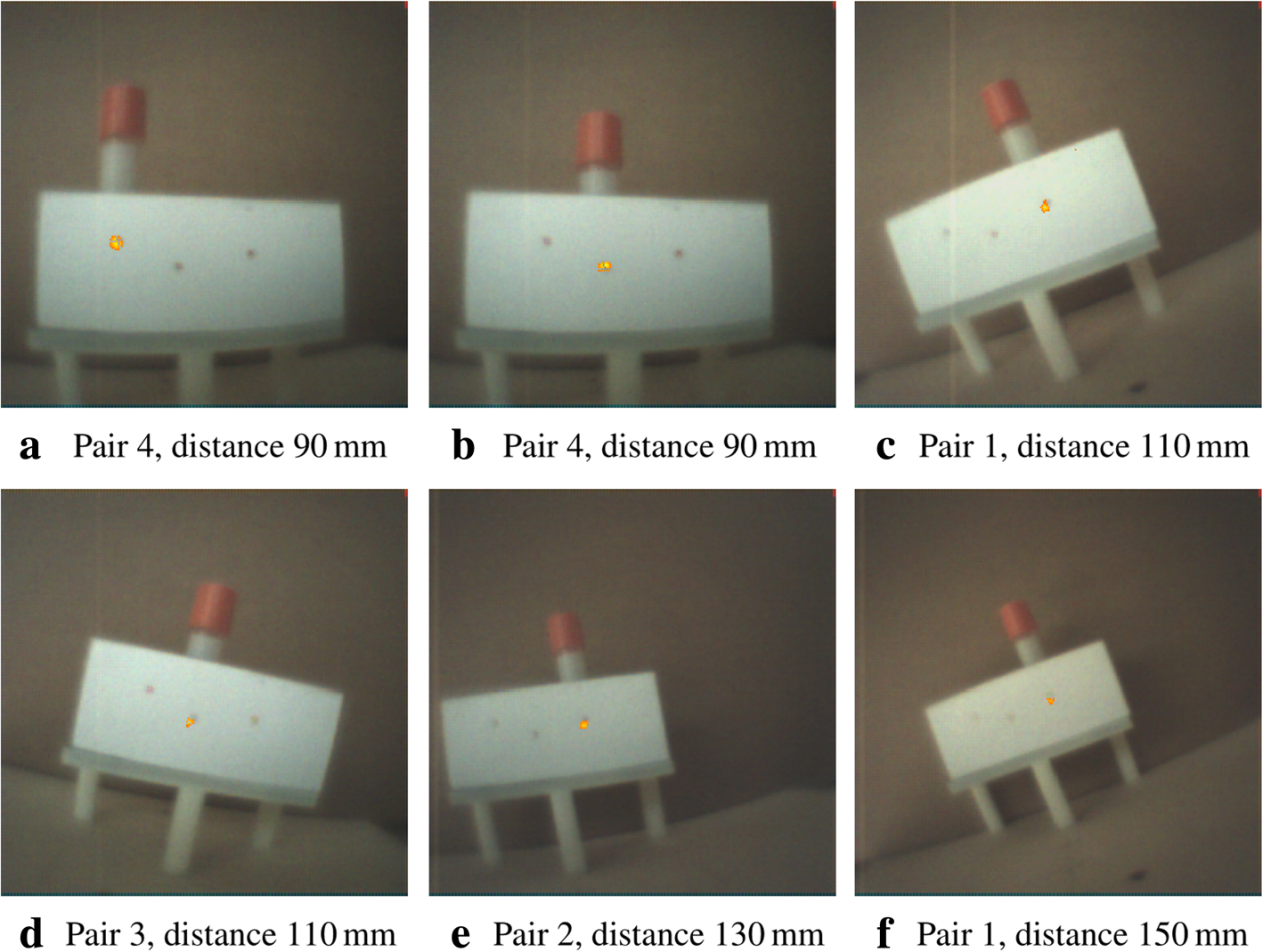
A collection of different augmentation results on a neutral target, reusing the respective stored extrinsic calibration of each pair (**a**-**f**)

**Fig. 20:**
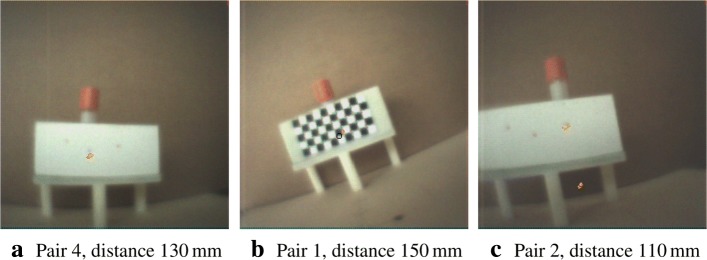
Some error cases. The exposure time for these measurements is 16 s. **a** Overestimating the true distance: estimated distance 150 mm, the augmentation is slightly off. **b** Underestimating the true distance: estimated distance 110 mm, the discrepancy is visible. **c** Pathologic case: a ghostly (false) augmentation in the foreground due to high scattering not properly filtered

## Discussion

Combining a multi-pinhole collimator with axis-aligned camera/pinhole pairs to augment optical camera images using gamma detector data is promising, as can be seen in the “Results” section. Based on the mathematical foundation and the conducted experiments, we show that the augmentation is valid for the tested depth estimates between 90 and 150 mm. Given the error plots, we conclude that depth estimates in the range of 70 to 250 mm are below the error bound and remain valid as well. The workspace setup of the surgeon is thus not limited by a too narrow distance regime of the device with respect to the patient’s neck. This warrants further development of the method and the device in order to gain in augmentation accuracy. As the background signal from the tracer near the injection site is high, and the expected accumulation in the lymph nodes rather low compared to the point-like source activities of our targets, more experiments addressing this issue need to be done to assess the approach for general SNB staging. In the case of HNSCC, the injection site is usually located at the tongue and thus away from the biopsy site. The discrimination of active over- and underlying tissue (so-called warm background) and potential sentinel nodes can be supported by inspecting the actual anatomy in case of an incision. However, for the initial placement of the biopsy tools in such an environment, or in case lymph nodes are positioned exactly above and below each other, specific targets need to be designed and tested with our method. Low activity deposits of the initially injected doses in the lymphatics and tracer absorbing layers are other difficulties. As we do not have real-time update requirements, increasing the integration time of the device to collect more photons, and thus to get a better signal-to-background ratio, remedies these problems. Building an improved collimator to constrain unwanted background photons is an important next step. Device integration is in strong focus of the development. Challenges in image processing remain to properly filter and display the true activities (i.e., sentinel lymph nodes). Furthermore, the quality of the augmentation depends not only on a good initial joint calibration but also on mechanical stability and the exactitude of the assembly process (e.g., axis alignment). Nevertheless, even with limited micro-manufacturing abilities (some cameras exhibit pronounced rotations and tilts), the augmentations remain below or near our defined error bound. This shows the flexibility of the method. Finally, more synthetic tests with specific phantoms and different dosages as well as in vivo animal experiments need to be conducted to assess the sensitivity of our approach.

## Conclusions

The strong dependence on preoperative imaging and the rather basic intraoperative orientation provided by one-dimensional audio-based gamma detectors are limiting factors for the successful application of sentinel lymph node biopsy (SNB). In HNSCC, a more targeted SNB enables a more reliable post-operative histopathologic staging, and therefore a more effective analysis of potential tumor spreading. Breast cancer and melanoma staging based on SNB face similar challenges. Our approach might therefore also be applicable in these domains and could provide a step forward for SNB in general.

## Appendix

### IV. Augmentation algorithm

How an actual optical camera image augmentation loop, based on the above principles, might be implemented is presented in this section. For each new depth estimate, we have to adapt $S_{p^{\prime }}$ and $S_{c^{\prime }}$ accordingly (*cf.* “New scaling matrices” section and Eq. (9)). CLUT is a look-up table to match intensities and colors.


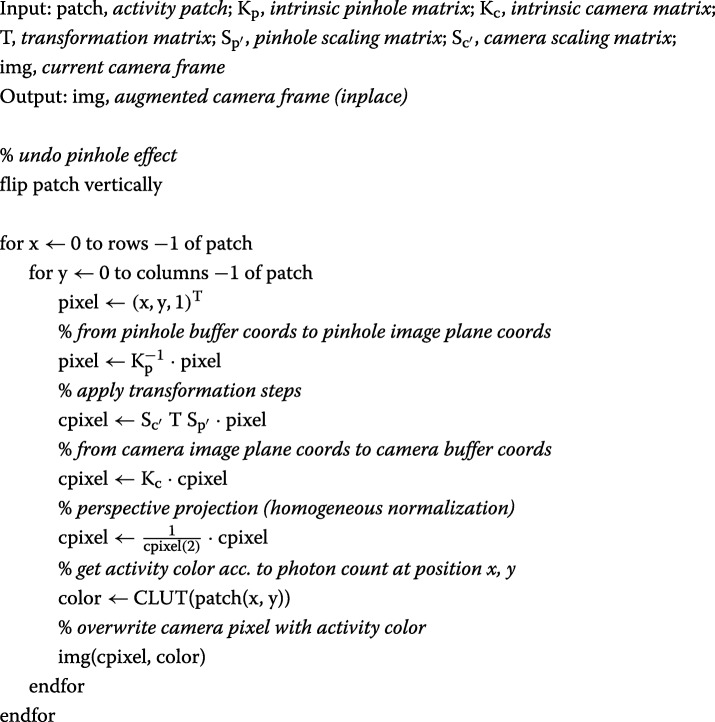


## Abbreviations

99mTc: Technetium 99m (nuclear medicine radioactive tracer)
ABS: Acrylonitrile butadiene styrene 404 (3d printing material)
AR: Augmented reality
CMOS: Complementary metal-oxide-semiconductor
cN0-neck: Clinically negative neck
fhSPECT: Freehand single photon emission computed tomography
GOSTT: Guided intraoperative scintigraphic tumor targeting
HNSCC: Head and neck squamous cell carcinoma
IAEA: International Atomic Energy Agency
ND: Neck dissection
SLN: Sentinel lymph node
SNB: Sentinel lymph node biopsy
SPECT/CT: Single photon emission computed tomography/computed tomography

## Footnotes

1
http://www-pub.iaea.org/books/IAEABooks/10661/ Guided-Intraoperative-Scintigraphic-Tumour-Targeting-GOSTT
